# Short-term sugar stress induces compositional changes and loss of diversity of the supragingival microbiota

**DOI:** 10.1080/20002297.2023.2189770

**Published:** 2023-03-21

**Authors:** Christine Lundtorp Olsen, Merete Markvart, Vincent Frederik Dahl Vendius, Christian Damgaard, Daniel Belstrøm

**Affiliations:** a Section for Clinical Oral Microbiology, Department of Odontology, Faculty of Health and Medical Sciences, University of Copenhagen, Copenhagen, Denmark; b ADM Denmark A/S, Hundested, Denmark; c Section for Oral Biology and Immunopathology, Department of Odontology, Faculty of Health and Medical Sciences, University of Copenhagen, Copenhagen, Denmark

**Keywords:** Oral microbiota, dental plaque, 16S rDNA, sugar stress, clinical trial

## Abstract

Frequent intake of free sugars is a major risk factor for dental caries, but the immediate influence of sugar intake on the supragingival microbiota remains unknown. We aim to characterize the effect of 14 days of sugar rinsing on the supragingival microbiota. Forty orally and systemically healthy participants rinsed their mouth with a 10% sucrose solution, 6–8 times a day, for 14 days, followed by 14 days without sugar stress. Supragingival plaque samples were collected at baseline, and after 14, and 28 days. The supragingival microbiota was analyzed using 16S rDNA sequencing. Taxonomic classification was performed using the Human Oral Microbiome Database. After 14 days of sugar stress induced by the daily sugar rinses, a significant loss of α-diversity (*p* = 0.02) and a significant increase in the relative abundance of *Actinomyces* (6.5% to 9.6%, *p* = 0.006) and *Corynebacterium* (6.2% to 9.1%, *p* = 0.03) species were recorded. In addition, a significant decrease in *Streptococcus* (10.3% to 6.1%, *p* = 0.001) species was observed. Sugar-mediated changes returned to baseline conditions 14 days after the last sugar rinse. The present study shows that temporary sugar stress induces loss of diversity and compositional changes to the supragingival microbiota, which are reversible if oral care is maintained.

## Introduction

Decades ago, the controversial Vipeholm Study demonstrated that the development of dental caries is strongly influenced by the intake of free sugars [1]. Thus, today free sugars are accepted as the most important dietary risk factor for the development of dental caries [2,3]. The association appears to be dose-dependent, with observations of a higher incidence of dental caries among individuals with an intake of free sugars>10% of total energy intake, compared to individuals with an intake of free sugars<10% of total energy intake [4].

Frequent intake of free sugars is an external perturbation to the oral ecosystem. Accordingly, cross-sectional data show that the salivary microbiota differs significantly in individuals with different levels of sugar intake [5]. The supragingival microbiota in individuals with a high sugar intake is characterized by less diversity and a higher abundance of *Actinomyces*, *Rothia, Lactobacillus, Veillonella*, and *Streptococcus* species compared to individuals with a low sugar intake [5,6]. Previous data highlight that the acidogenic and aciduric members of the oral microbiota thrive when the oral ecosystem is exposed to carbohydrates, but interventional studies are needed to reveal the direct impact of frequent sugar intake on the oral ecosystem.

With this in mind, we have recently tested the impact of rinsing with a 10% sucrose solution, 6–8 times per day, for 14 days on the salivary microbiota in orally healthy individuals [7]. We found that the frequent intake of sucrose induced distinct compositional changes to the salivary microbiota, with a substantial increase of *Streptococcus* species, which was completely reversed, when the study-induced sugar stress was removed [7]. While our data on the salivary microbiota confirm the significant impact of frequent sugar intake on the oral ecosystem, they provide no information about the effect of sugar stress on the supragingival microbiota, which is the central pathologic factor in dental caries. Consequently, if we are to evaluate the effect of sugar stress in the context of dental caries, longitudinal data of frequent sugar intake on the supragingival microbiota are needed.

Therefore, the present study aimed to characterize how short-term sugar stress affects the supragingival microbiota. We tested the hypothesis that 14 days of sugar stress induces loss of diversity and compositional changes in the supragingival biofilm, but that these changes are reversible and return to baseline conditions when the sugar stress is terminated.

## Materials and methods

### Study design

**Figure 1.**
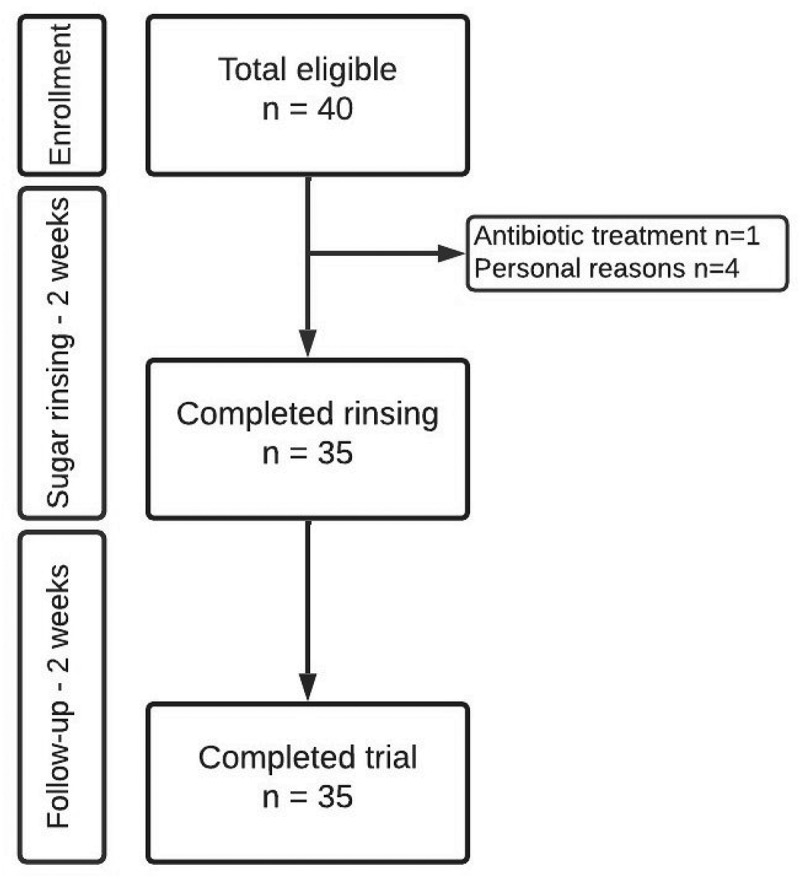
Flowchart of the study.

From November to December 2021, we performed a longitudinal, interventional study, with a total duration of 28 days, at the Department of Odontology, University of Copenhagen, Denmark (Figure 1). The procedures of the sugar stress trial have been described previously [7]. In brief, the trial was comprised of a perturbation period of 14 days, during which an oral sucrose rinse was performed every second hour (approx. 6–8 times per day), followed by a resolution period of 14 days, where sugar rinsing was discontinued. Throughout the entire study, participants were allowed to perform regular oral care. All participants signed informed consent. The study was approved by the regional ethical committee (H-21003295), registered at ClinicalTrials.gov (UCPH_01_005), and reported to the local data authorization of the Faculty of Health and Medical Sciences, University of Copenhagen (514–0434/19–3000).

### Study population

The study population consisted of a total of 40 individuals with good oral and systemic health, who were originally enrolled as the placebo group in a large randomized clinical trial (unpublished data). The size of the study population was decided based on a power calculation using data from our previously published paper [7]. Specifically, data showed that *n* = 20 was sufficient to detect a 70% increase in *Streptococcus* species in saliva after 14 days of sugar stress. Because we expected the impact of sugar stress to be approximately 50% less on the supragingival microbiota, we extrapolated this to mean that a sample size of *n* = 35 was needed. With an expected dropout of 10%, 40 participants were enrolled. Inclusion criteria: age 18–35 years. Exclusion criteria: systemic diseases requiring medical treatment, current smokers, treatment requiring oral diseases (dental caries, periodontitis), generalized gingivitis (BOP<15%), pregnancy, and use of antibiotics in the past three months.

### Clinical examination

Clinical examinations were completed at baseline, day 14, and day 28 (±2 days) by the same examiner (CLO), and have been described in detail elsewhere [8]. In brief, plaque levels and bleeding on probing (BOP) were recorded at six sites per tooth for the entire dentition (third molar excluded). Plaque levels were registered using SUNSTAR G.U.M®^MD^ RED-COTE®^MD^ disclosing tablets, and scored from 0 to 5 by use of the Modified Quigley and Hein index [9], while BOP was scored from 0 to 2 [10].

### Collection of samples

Supragingival plaque samples were collected from the buccal surface of the 1^st^ quadrant before clinical examinations (at baseline, day 14, and day 28). Samples were collected throughout the day, but great effort was made to collect all samples from each participant at the same time during the three trial days. To ensure sufficient sample material, participants did not perform oral care on the day of sampling. Samples were pooled and vortexed in 1 mL saline and put into temporary storage at −18°C. Within 8 h of sample collection samples were stored at −80°C to await further analyses.

### Sucrose solution

Production of the sucrose solution and the rinsing protocol has been described previously [7]. In brief, participants were instructed to rinse with the sucrose solution for at least half a minute every second hour (6–8 times per day). Furthermore, participants were informed not to consume any food or beverages 15 min after rinsing.

### DNA extraction, library preparation, and DNA sequencing

DNA extraction, library preparation, and 16S sequencing followed the same protocol as in our previous studies, which has been described previously [7,8]. In brief, we targeted the V1-V3 region of the 16S gene by use of MiSeq (Illumina, San Diego, California) [7,8]. After quality control, only samples with>8000 reads were included in the downstream analysis. For this project, no samples failed and all samples were therefore included in the analyses.

### Bioinformatics and statistics

Bleeding and plaque index was compared between sampling times in Microsoft excel v. 2016 using *t*-test and one-factor ANOVA testing at a 95% confidence level.

Bioinformatic processing of sequence data was performed as previously described [7,8]. In brief, 16S rRNA data were taxonomically referenced with the Human Oral Microbiome RefSeq database (HOMD) v. 15.2 [11], and all further analyses made using R v.4.1.0 through the Rstudio IDE ampvis package v.2.7.8 [12]. The relative abundance of the supragingival microbiota was compared between sampling times, with data being corrected by Benjamini-Hocherg correction [13]. For these analyses, an adjusted p-value≤0.05 was considered significant. In addition, microbial composition data were analyzed using Linear discriminant analysis Effect Size (LEfSe) [14].

Alpha diversity was calculated by Shannon index and Simpson index and compared between sampling times using paired *t-*tests testing at a 95% confidence level. Alpha diversity was visualized by density plots. Beta diversity was tested using principal component analysis (PCA).

## Results

### Background and clinical data

Thirty-five subjects completed the trial, while five subjects dropped out during the trial period due to antibiotic prescription (*n* = 1) and personal reasons (*n* = 4). All dropouts happened within the first two weeks and were therefore excluded from further analyses. Women dominated the study population, as did dental professionals (Table 1). The remaining participants were friends of, or in a relationship with a dental professional. During the short-term sugar stress phase, the average plaque index decreased significantly from a mean of 1.72 to 1.57 (*p* = 0.004) and remained low in the subsequent 14 days (mean = 1.55) (Table 2). Sugar stress had no significant impact on neither the bleeding index (*p* = 0.3) nor bleeding percentage (*p* = 0.2) (Table 2).

**Table 1.** Background information on the study population. Dropouts were not included as they dropped out within the first 14 days and were thus excluded from further analysis.

	Participants
Sex (female/male)	26/9
Age (mean, range)	23.4 (19-30)
Dental professions*	25/35

*Dentists, dental students.

**Table 2.** Clinical endpoints measured by means and range of plaque index, bleeding index, and percentage of bleeding on probing (BOP%).

	Mean plaque index	Mean bleeding index	BOP%
Baseline	1.72 (1.07–2.40)	0.03 (0–0.10)	2.92 (0–10.12)
Week 2	1.57* (1.01–2.13)	0.03 (0–0.14)	3.40 (0–13.69)
Week 4	1.55** (1.01–2.13)	0.03 (0–0.09)	3.50 (0–9.52)

*Significant difference between baseline and week 2.

**Significant difference between baseline and week 4.

### Sequencing metadata

DNA extraction and sequencing library preparation was successful for 105/105 sample analyses (100%) and yielded between 42.602 and 202.974 DNA reads after QC and bioinformatic processing. Thus, a total of 7.98 million reads (42.602 reads per sample) were included. A total of 2.998 unique OTUs could be identified, belonging to 93 different bacterial genera (mean: 65, range: 55–76) and 335 different bacterial species (mean: 203, range: 154–243). Consequently, 92.96% and 60.81% of sequences could be identified at genus and species levels, respectively. The mean alpha diversity across all 105 samples, as determined by the Shannon index, was 4.07.

### Short-term sugar stress induces compositional changes to the supragingival microbiota

**Figure 2.**
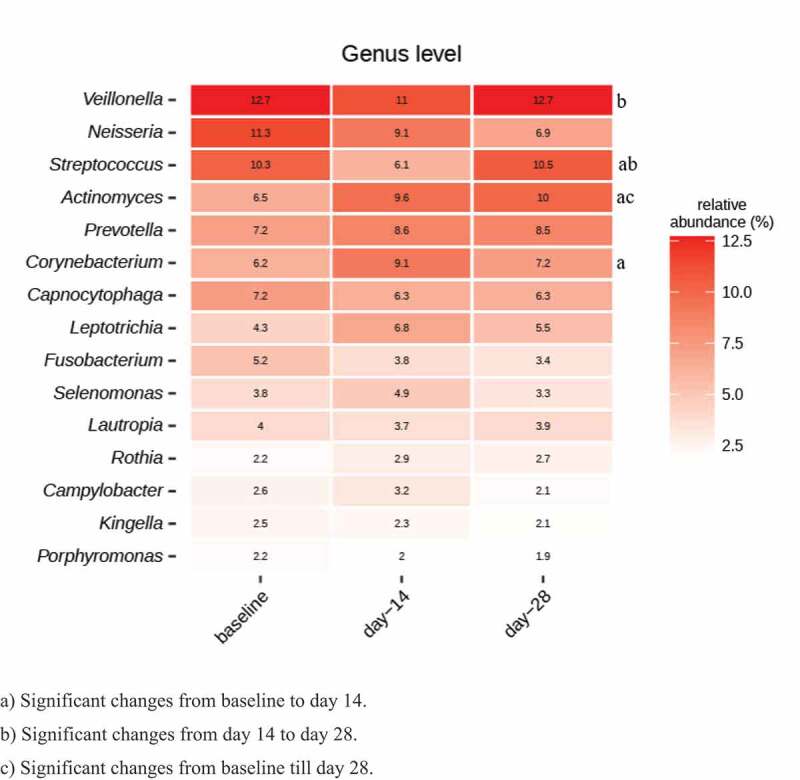
Impact of sugar stress on predominant microbiota. Relative abundance of 15 predominant genera expressed as the mean value. a) Significant changes from baseline to day 14. b) Significant changes from day 14 to day 28. c) Significant changes from baseline till day 28.

Sugar stress caused a significant decrease in the relative abundance of *Streptococcus* from 10.3% to 6.1% (*p* = 0.001), together with a significant increase in the relative abundance of *Actinomyces* from 6.5% to 9.6% (*p* = 0.006) and *Corynebacterium* from 6.2% to 9.1% (*p* = 0.03) (Figure 2). Compositional changes of *Streptococcus* (baseline: 10.3%, day 28: 10.5%) and *Corynebacterium* (baseline: 6.2%, day 28: 7.2%) species were almost completely reversed at day 28 (Figure 2). However, the relative abundance of *Actinomyces* (baseline: 6.5%, day 28: 9.9%) remained significantly elevated (*P* = 0.003).

**Figure 3.**
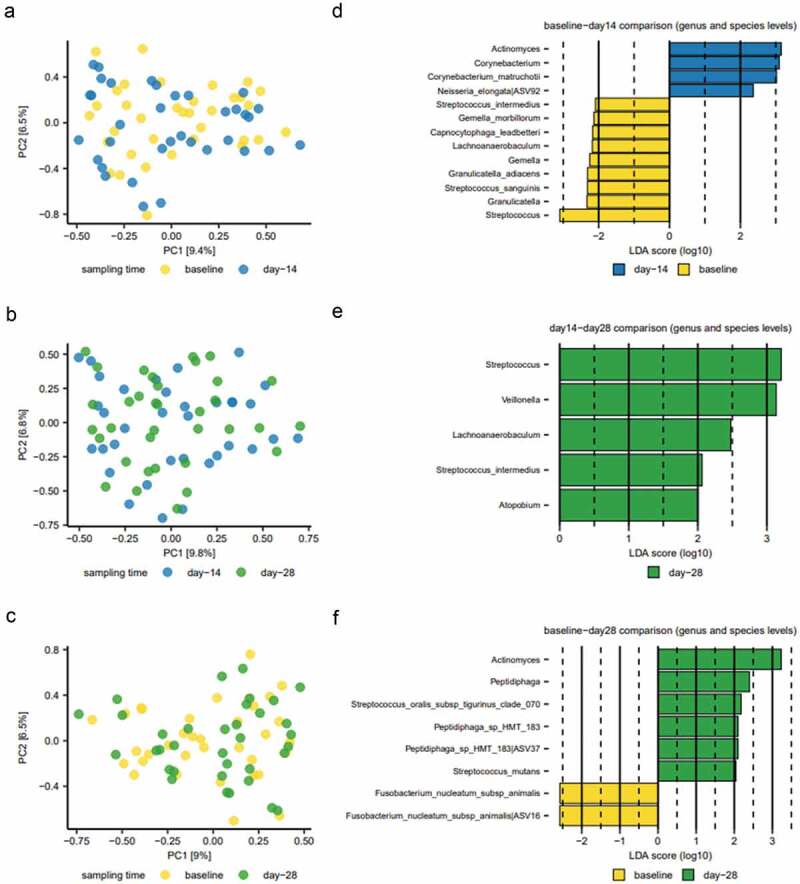
Compositional changes induced by sugar stress. Principal component analysis (PCA) is expressed by the two most decisive components (PC1 and PC2), which covered approximately 16% of the variation of the dataset. **(A)** baseline vs. week 2. **(B)** week 2 vs week 4. **(C)** baseline vs week 4. Linear discriminant analysis Effect Size (LEfSe) analysis expressed by significant genera and species at baseline vs. week 2 **(D)**, week 2 vs week 4 **(E)**, and baseline vs week 4 **(F)**.

Principal component analysis (PCA) revealed a random distribution of baseline samples and samples collected on day 14 (after two weeks of sugar stress) (Figure 3A). Likewise, no clustering of day 14 and day 28 samples was observed (Figure 3B). Finally, samples collected at baseline and samples collected 14 days after discontinuation of sugar stress (week 4) showed random distribution (Figure 3C).

Using Linear discriminant analysis Effect Size (LEfSe) analysis, 11 bacterial genera and 15 bacterial species, were identified as differing significantly between sampling times (Figure 3D–F). *Actinomyces* and *Corynebacterium* species were significantly associated with samples collected after 14 days of sugar stress (Figure 3D), while *Streptococcus* species were significantly associated with baseline samples and samples collected 14 days after resolution of the sugar stress (Figure 3D and E). *Actinomyces* species continued to be significantly associated with samples collected on day 28 (Figure 3F).

### Short-term sugar stress causes a loss in diversity in the supragingival microbiota

**Figure 4.**
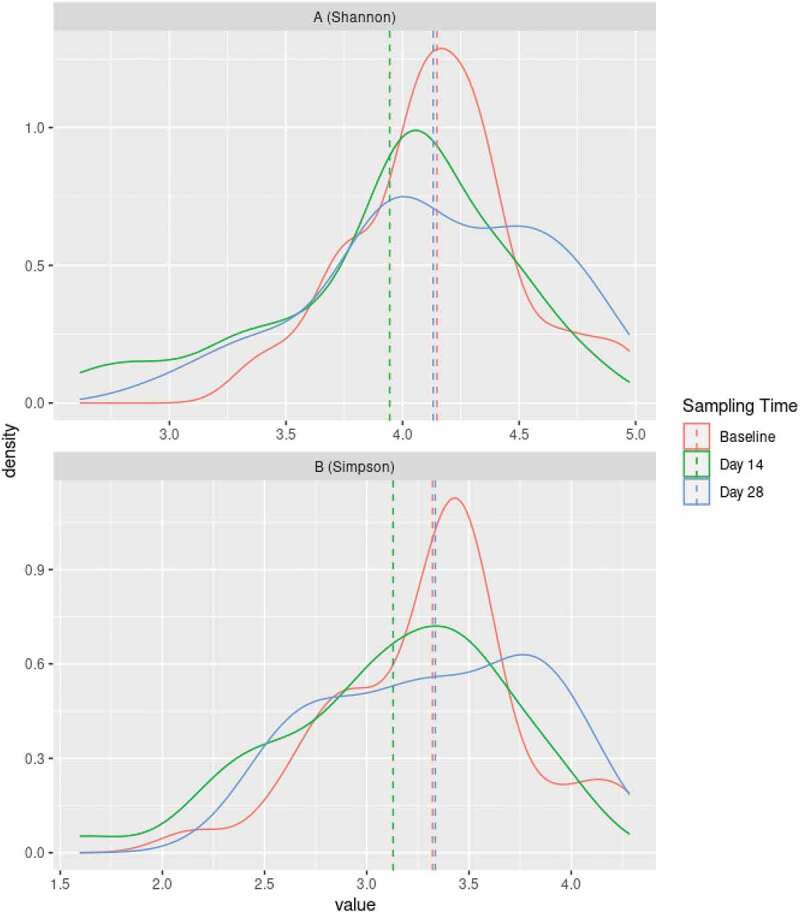
Changes in diversity induced by sugar stress. Density plots based on Shannon index **(A)** and Simpson index **(B)** at baseline (red line), two weeks after sugar stress (green line), and two weeks after discontinuation of sugar stress at week 4 (blue line).

Density plots of α-diversity (Shannon index) showed a significant loss of diversity (*p* = 0.02) from baseline (red line) to week 2 (green line), which was reversed two weeks after discontinuation of the sugar stress (blue line) (Figure 4A). Likewise, density plots using the Simpson index illustrated a loss of diversity from baseline (red line) to week 2 (green line), which was also reversed at week 4 (blue line). Data from the Simpson index were borderline significant (*p* = 0.08) (Figure 4B).

## Discussion

The purpose of the present study was to characterize the effect of short-term sugar stress on the supragingival microbiota. We tested the hypothesis that 14 days of sugar stress induce a loss of diversity and compositional changes in the supragingival biofilm, which are completely reversible. To the best of our knowledge, this is the first interventional study to characterize the longitudinal impact of short-term sugar stress on the supragingival microbiota.

The main finding is that short-term sugar stress induces a significant loss of α-diversity in the supragingival microbiota (Figure 4A). This finding is in accordance with the ecological plaque hypothesis [15], in which frequent sugar consumption is considered the critical external perturbation, as this promotes favorable living conditions for aciduric and acidogenic species at expense of health-associated species, thereby leading to loss of diversity and potentially to the development of dental caries [16,17]. The finding is further supported by previous studies comparing individuals with different levels of sugar intake, which also found significantly lower diversity among individuals with a high sugar intake [6]. Interestingly, cross-sectional data have previously shown that samples from surfaces with dental caries are characterized by lower diversity compared to samples from healthy supragingival sites [18]. Likewise, cross-sectional data have shown significantly lower microbial diversity in the saliva of individuals with dental caries compared to orally healthy individuals [19]. As such, the present longitudinal data reinforce the central role of frequent sugar intake in the pathogenesis of dental caries, as frequent sugar intake in and of itself induces a significant loss of bacterial diversity, which remains a hallmark event in the development and progression of a caries lesion [18,20].

As expected, sugar stress had a significant impact on the composition of the supragingival microbiota (Figures 2, 3D and E). Surprisingly, sugar stress significantly decreased the relative abundance of *Streptococcus* species (from 10.3% to 6.1%, *p* = 0.001). This was highly unexpected, as most members of the genus *Streptococcus* are known to be proficient in carbohydrate metabolism, why *Streptococcus* species have also traditionally been associated with especially initial stages of dental caries [21,22]. Indeed, we recently demonstrated that short-term sugar stress induces a significant increase in *Streptococcus* species in saliva [7], which confirms the capability of *in-vivo* carbohydrate consumption in the oral cavity by *Streptococcus* species. It is therefore interesting that sugar stress has an opposite effect on *Streptococcus* species in saliva and supragingival plaque, which may reflect the difference between the planktonic living conditions in saliva versus the supragingival biofilm. Recent *in-vivo* studies on adults using next-generation sequencing have not demonstrated any significant difference in the abundance of *Streptococcus* species between healthy supragingival sites and supragingival sites with dental caries [18,23,24]. Altogether, this calls into question the hitherto critical role that *Streptococcus* species has been assigned in the pathogenesis of dental caries [18,23,24].

*Streptococcus mutans* has traditionally been perceived as the primary cariogenic pathogen. In the present study, short-term sugar stress had no significant impact on the relative abundance of *S. mutans* in the supragingival plaque, which is no surprise as the participants had no treatment-requiring cavities, which could favor the growth of *S. mutans*. It is important to remember that the composition of the oral microbiota seems to change as dental caries progresses [18], with *S. mutans* being associated with the advanced caries lesion [21,22,25] and have been found in increased levels in individuals with a high prevalence of dental caries [26]. To the best of our knowledge, only one study has used next-generation sequencing to compare the microbiota characterized by caries experience using the DMFT-index [18], in which no difference in the abundance of *S. mutans* between groups was found. Likewise, a recent 16S-based study reported that although *S. mutans* occurred more frequently in the caries group, *S. mutans* was absent in more than 50% of the samples from adolescents with dental caries [24]. Thus, data from the present study add to the growing body of literature, which argues that from a microbiological perspective dental caries is the consequence of a continual ecological imbalance in the supragingival microbiota fueled by frequent sugar intake and inadequate oral hygiene, which can occur with and without the presence of *S. mutans.*

Present data indicate that members of the supragingival microbiota, such as *Actinomyces* and *Corynebacterium* species outperform *Streptococcus* when exposed to frequent sugar intake in otherwise healthy conditions. This points to their potentially critical role in the transition from symbiosis to sugar-mediated dysbiosis. Similarly, data demonstrated that sugar stress instigated a significant increase in the abundance of *Actinomyces* species (Figures 2, 3D and F). This is in line with previous findings, where *Actinomyces* species have been found in higher abundance in individuals with dental caries compared to healthy individuals [16,27]. Certain non-aciduric members of the oral microbiota, such as *Corynebacterium* and *Granulicatella* species, have been reported to associate with caries-associated dental plaque [16], and it is therefore noteworthy that sugar stress induced a significant increase in the abundance of *Corynebacterium* species from 6.2% to 9.1%, *p* = 0.03 (Figures 2, 3D). As such, the present findings are consistent with the contemporary assumption that dental caries is the consequence of a complicated shift in the ecological balance, which affects multiple species.

Importantly, data from the present study do not provide any information about dental caries, as the supragingival samples were collected from healthy surfaces. However, data highlight that frequent sugar intake, which is considered a risk factor for dental caries, appears to favor the growth of *Actinomyces* and *Corynebacterium* species at the expense of the growth of *Streptococcus* species in supragingival plaque. Thus, these data suggest *Actinomyces* and *Corynebacterium* species as an alternative to *Streptococcus* species, as candidate targets for caries prevention strategies, such as probiotics and antibacterial compounds.

Notably, the sugar-mediated changes to the supragingival microbiota appeared to be almost completely reversible, as evaluated by the relative abundance of predominant genera (Figure 2), principal component analysis (Figure 3), and α-diversity (Figure 4). As such, the present data show the ability of the oral microbiota to return to baseline conditions after being exposed to a transient perturbation such as sugar stress. Interestingly, this finding corresponds with data on recovery profiles from the salivary microbiota after treatment with antibiotics [28] and data from our previous study on sugar stress and the salivary microbiota [7]. Collectively, these data demonstrate that the oral microbiota is characterized by a high degree of resilience, i.e. colonization memory, after being exposed to external perturbations, as long as the duration of these are temporary [29].

Clinical data from the present study showed a high level of self-performed oral hygiene throughout the study, as evaluated by consistently low plaque- and bleeding indexes (Table 2). In fact, the plaque index decreased significantly during sugar stress (*p* = 0.004), which is in line with participants reporting an increased need for oral hygiene due to an increased feeling of plaque accumulation during sugar rinsing. While the plaque index decreased, sugar stress did not significantly impact the bleeding index and bleeding percentage (*p* = 0.3 and *p* = 0.2, respectively) (Table 2). Bleeding is of particular interest as increasing evidence suggests that a high sugar intake impacts the inflammatory response [29]. However, as sugar stress only lasted two weeks in the present trial, while oral hygiene procedures improved, bleeding parameters remained unaffected. Collectively, data from the present study underline that if there are no dental carious lesions present in the oral cavity, and sufficient oral care is performed, transient sugar stress is only able to induce reversible changes to the oral microbiota. Thus, data reinforce that a high level of self-performed oral care remains the best preventive regimen against the development of dental caries. However, the present study cannot answer whether persistent sugar stress could induce irreversible changes despite regular oral hygiene. Ideally, this needs to be elucidated in future studies, which for ethical reasons might be difficult to conduct.

Some limitations apply to the present study, including the homogeneous study population comprised of young and healthy individuals with a high standard of oral hygiene (Tables 1–2), lowering the external validity of the data presented. However, from an ethical point of view, it was only acceptable to perform temporary sugar stress in orally healthy individuals with a high level of oral care. Moreover, systemic diseases [30] and smoking [31], have been documented to impact the composition of the oral microbiota, which is why individuals with these characteristics were excluded, as these parameters would potentially act as a confounder on the effect of sugar stress. Therefore, we deliberately chose a healthy and homogeneous study population. In the present study, we used 16S sequencing to study the effect of sugar stress. While 16S sequencing provides valuable taxonomic information, information about the underlying biological mechanisms remains uncovered. Consequently, future studies using metagenomics and metatranscriptomics to study the effect of sugar stress on the oral microbiota are highly warranted.

In conclusion, the present study shows that temporary sugar stress induces changes in the supragingival microbiota, characterized by a loss of diversity, an increase in *Actinomyces* and *Corynebacterium* species, and a decrease in *Streptococcus* species, which are all completely reversible if the sugar stress is terminated. Thus, we suggest that a loss of diversity and a decrease in *Streptococcus* abundance are the hallmarks of sugar-mediated dysbiosis, which, if left undisturbed for a prolonged time, may be the first step toward the development of dental caries. Future studies, presumably in well-designed animal models, are needed to clarify the duration of sugar stress needed to induce clinical signs of dental caries.

## Data Availability

Raw sequences have been deposited in European Nucleotide Archive (ENA, www.ebi.ac.uk, accessed on 7 March 2023) with the accession number PRJEB58919.

## References

[1] Gustafsson BE, Quensel CE, Lanke LS, et al. The Vipeholm dental caries study; the effect of different levels of carbohydrate intake on caries activity in 436 individuals observed for five years. Acta Odontol Scand. 1954;11(3–4):232–9. DOI:10.3109/00016355308993925 13196991

[2] Sheiham A. Dietary effects on dental diseases. Public Health Nutr. 2001;4(2b):569–591. 11683551 10.1079/phn2001142

[3] Diet, nutrition and the prevention of chronic diseases: report of a joint WHO/FAO expert consultation. WHO Technical Report Series, No. 916. Geneva: World Health Organization; 2003. 12768890

[4] Moynihan PJ, Kelly SA. Effect on caries of restricting sugars intake: systematic review to inform WHO guidelines. J Dent Res. 2014;93(1):8–18. 24323509 10.1177/0022034513508954 PMC3872848

[5] Esberg A, Haworth S, Hasslöf P, et al. Oral microbiota profile associates with sugar intake and taste preference genes. Nutrients. 2020;12(3):681. 32138214 10.3390/nu12030681 PMC7146170

[6] Angarita-Díaz MDP, Fong C, Bedoya-Correa CM, et al. Does high sugar intake really alter the oral microbiota?: a systematic review. Clin Exp Dent Res. 2022;8(6):1376–1390. 35946056 10.1002/cre2.640 PMC9760141

[7] Lundtorp-Olsen C, Enevold C, Juel Jensen CA, et al. Impact of probiotics on the salivary microbiota and salivary levels of inflammation-related proteins during short-term sugar stress: a randomized controlled trial. Pathogens. 2021;10(4):392. 33805894 10.3390/pathogens10040392 PMC8064398

[8] Lundtorp-Olsen C, Enevold C, Twetman S, et al. Probiotics do not alter the long-term stability of the supragingival microbiota in healthy subjects: a randomized controlled trial. Pathogens. 2021;10(4):391. 33805208 10.3390/pathogens10040391 PMC8064340

[9] Lobene RR, Soparkar PM, Newman MB. Use of dental floss. Effect on plaque and gingivitis. Clin Prev Dent. 1982;4(1):5–8. 6980082

[10] Saxton CA, van der Ouderaa FJ. The effect of a dentifrice containing zinc citrate and triclosan on developing gingivitis. J Periodontal Res. 1989;24(1):75–80. 2524573 10.1111/j.1600-0765.1989.tb00860.x

[11] Escapa IF, Chen T, Huang Y, et al. New insights into human nostril microbiome from the expanded human oral microbiome database (eHOMD): a resource for the microbiome of the human aerodigestive tract. mSystems. 2018;3(6):7–18. 10.1128/mSystems.00187-18 PMC6280432 30534599

[12] Albertsen M, Karst SM, Ziegler AS, et al. Back to Basics–the influence of DNA extraction and primer choice on phylogenetic analysis of activated sludge communities. PLoS ONE. 2015;10(7):e0132783. 26182345 10.1371/journal.pone.0132783 PMC4504704

[13] Hochberg Y, Benjamini Y. More powerful procedures for multiple significance testing. Stat Med. 1990;9(7):811–818. 2218183 10.1002/sim.4780090710

[14] Segata N, Izard J, Waldron L, et al. Metagenomic biomarker discovery and explanation. Genome Biol. 2011;12(6):R60. DOI:10.1186/gb-2011-12-6-r60 21702898 PMC3218848

[15] Marsh PD. Microbial ecology of dental plaque and its significance in health and disease. Adv Dent Res. 1994;8(2):263–271. 7865085 10.1177/08959374940080022001

[16] Valm AM. The structure of dental plaque microbial communities in the transition from health to dental caries and periodontal disease. J Mol Biol. 2019;431(16):2957–2969. 31103772 10.1016/j.jmb.2019.05.016 PMC6646062

[17] Pitts NB, Zero DT, Marsh PD, et al. Dental caries. Nat Rev Dis Primers. 2017;3(1):17030. DOI:10.1038/nrdp.2017.30 28540937

[18] Xiao C, Ran S, Huang Z, et al. Bacterial Diversity and community structure of supragingival plaques in adults with dental health or caries revealed by 16S pyrosequencing. Front Microbiol. 2016;7:1145. 27499752 10.3389/fmicb.2016.01145 PMC4956651

[19] Belstrøm D, Holmstrup P, Fiehn NE, et al. Salivary microbiota in individuals with different levels of caries experience. J Oral Microbiol. 2017;9(1):1270614. DOI:10.1080/20002297.2016.1270614 28326153 PMC5328370

[20] Du Q, Fu M, Zhou Y, et al. Sucrose promotes caries progression by disrupting the microecological balance in oral biofilms: an in vitro study. Sci Rep. 2020;10(1):2961. DOI:10.1038/s41598-020-59733-6 32076013 PMC7031525

[21] Struzycka I. The oral microbiome in dental caries. Pol J Microbiol. 2014;63(2):127–135. 25115106

[22] Takahashi N, Nyvad B. The role of bacteria in the caries process: ecological perspectives. J Dent Res. 2011;90(3):294–303. 20924061 10.1177/0022034510379602

[23] Jiang Q, Liu J, Chen L, et al. The oral microbiome in the elderly with dental caries and health. Front Cell Infect Microbiol. 2018;8:442. 30662876 10.3389/fcimb.2018.00442 PMC6328972

[24] Havsed K, Stensson M, Jansson H, et al. Bacterial composition and metabolomics of dental plaque from adolescents. Front Cell Infect Microbiol. 2021;11:716493. 34395316 10.3389/fcimb.2021.716493 PMC8362896

[25] Aas JA, Griffen AL, Dardis SR, et al. Bacteria of dental caries in primary and permanent teeth in children and young adults. J Clin Microbiol. 2008;46(4):1407–1417. DOI:10.1128/JCM.01410-07 18216213 PMC2292933

[26] Johansson I, Witkowska E, Kaveh B, et al. The microbiome in populations with a low and high prevalence of caries. J Dent Res. 2016;95(1):80–86. 26442950 10.1177/0022034515609554 PMC4700664

[27] Li X, Liu Y, Yang X, et al. The oral microbiota: community composition, influencing factors, pathogenesis, and interventions. Front Microbiol. 2022;13:895537. 35572634 10.3389/fmicb.2022.895537 PMC9100676

[28] Lazarevic V, Manzano S, Gaïa N, et al. Effects of amoxicillin treatment on the salivary microbiota in children with acute otitis media. Clin Microbiol Infect. 2013;19(8):E335–42. DOI:10.1111/1469-0691.12213 23565884

[29] Nyvad B, Takahashi N. Integrated hypothesis of dental caries and periodontal diseases. J Oral Microbiol. 2020;12(1):1710953. 32002131 10.1080/20002297.2019.1710953 PMC6968559

[30] Graves DT, Corrêa JD, Silva TA. The oral microbiota is modified by systemic diseases. J Dent Res. 2019;98(2):148–156. 30359170 10.1177/0022034518805739 PMC6761737

[31] Brook I. The impact of smoking on oral and nasopharyngeal bacterial flora. J Dent Res. 2011;90(6):704–710. 21558542 10.1177/0022034510391794

